# Effect of Dietary *n*‐3 Source on Rabbit Male Reproduction

**DOI:** 10.1155/2019/3279670

**Published:** 2019-12-16

**Authors:** Cesare Castellini, Simona Mattioli, Cinzia Signorini, Elisa Cotozzolo, Daria Noto, Elena Moretti, Gabriele Brecchia, Alessandro Dal Bosco, Giuseppe Belmonte, Thierry Durand, Claudio De Felice, Giulia Collodel

**Affiliations:** ^1^Department of Agricultural, Environmental, and Food Science, University of Perugia, Borgo XX Giugno 74, 06123 Perugia, Italy; ^2^Department of Molecular and Developmental Medicine, University of Siena, Policlinico Santa Maria alle Scotte, Viale Bracci 14, 53100 Siena, Italy; ^3^Department of Veterinary Medicine, University of Perugia, Via San Costanzo 4, 06123 Perugia, Italy; ^4^Institut des Biomolécules Max Mousseron (IBMM), UMR 5247, CNRS, Université de Montpellier, ENSCM, Montpellier, France; ^5^Child Neuropsychiatry Unit, Azienda Ospedaliera Universitaria Senese, Siena, Italy; ^6^Neonatal Intensive Care Unit, Azienda Ospedaliera Universitaria Senese, Siena, Italy

## Abstract

In the last two decades, the human sperm count linearly decreased in Western countries. Health problems, lifestyle, pollutants, and dietary behaviours are considered as the main risk factors, and the unbalance of dietary *n*‐6/*n*‐3 fatty acids is one of the most relevant. The aim of the present research is to study the effect of different dietary sources of *n*‐3 polyunsaturated fatty acids (PUFA) on reproductive traits using rabbit buck as the animal model. Fifteen rabbit bucks were assigned to three experimental groups: the control group, the FLAX group fed 10% extruded flaxseed, and the FISH group fed 3.5% fish oil for 110 days (50-day adaptation and 60-day experimental periods). Semen samples were collected weekly, whereas blood was collected every two weeks for the analytical determination of semen traits, oxidative status, fatty acid profiles, isoprostanes, neuroprostanes, and the immunocytochemistry of docosahexaenoic acid (DHA) and eicosapentaenoic (EPA) acid. At the end of the trial, the rabbits were killed and the testes were removed and stored for the analysis of fatty acid profile and immunocytochemistry. Results showed that dietary administration of *n*‐3 PUFA improved the track speed of the sperm and increased the *n*‐3 long-chain PUFA mainly confined in the sperm tail. Seminal plasma increased the thiobarbituric reactive substances (TBARs) by three times in the groups fed supplemental *n*‐3, whereas the F_2_-isoprotanes (F_2_-IsoPs) and F_4_-neuroprostanes (F_4_-NeuroPs) were lower and higher, respectively, in both supplemented groups than in the control. The testes and sperm showed a higher DHA and EPA distribution in rabbits from the *n*‐3 supplemented groups compared with the control. In conclusion, supplemental dietary *n*‐3 PUFA improved sperm motion traits and resulted in an enrichment of membrane fatty acid in the sperm and testes of the rabbits. However, such an increased amount of PUFA negatively affected the sperm oxidative status, which was mainly correlated with the generation of F_4_-NeuroPs with respect to F_2_-IsoPs. Accordingly, the latter cannot be considered a good marker of oxidation when diets rich in *n*‐3 PUFA are provided.

## 1. Introduction

In the last two decades, the sperm count has been progressively and linearly decreasing [1]; accordingly, in the next 30 years, a dramatic decrease in the fertility rate is expected. Many factors can reduce male fertility: chronic health problems, environmental pollutants, stress, and lifestyle, including dietary habits. Lipids play a crucial role in the structure and function of cells; among them polyunsaturated fatty acids (PUFA) represent about 30% to 50% of total fatty acids (FA) in the membrane of mammal spermatozoa. The body of literature has reported that the FA profile of the sperm membrane is different in men with asthenozoospermia compared with normospermic men [2].

Long-chain (LC, ≥20C) PUFA result from elongations and desaturations of essential FA: linoleic acid (LA, 18 : 2*n*‐6) and *α*-linolenic acid (ALA, 18 : 3*n*‐3). These FA are known to affect membrane behaviour and flexibility [3] and are eicosanoid precursors [4]. Testes and sperm have a characteristic lipid composition that is highly enriched in LC PUFA, predominantly docosapentaenoic acid (DPA*n*‐6, 22 : 5*n*‐6) in rats and other rodents and docosahexaenoic acid (DHA, 22 : 6*n*‐3) in humans [5]. PUFA accumulate in mammalian testes during puberty and are essential for sperm maturation, motility, and acrosome reaction [6]. They are incorporated into maturing germ cells by lysophosphatidic acid acyltransferase 3 [7]. An improper FA profile modifies the function of Sertoli cells as spermatogenesis supporters, influencing germ cell apoptosis [8]. Furthermore, during epididymal maturation, the lipid composition of the sperm membrane is remodelled and the saturation of FA increases from *caput* to the *cauda* epididymis, while the proportion of PUFA remains similar [9].

The current Western diet has an abundance of *n*‐6 with respect to *n*‐3 PUFA and the *n*‐6/*n*‐3 ratio is two to four times (10-20 : 1) higher than the requirement [10]. Dietary plans with different *n*‐6/*n*‐3 ratios may affect the sperm FA profile, physiology, and DNA integrity of the sperm and testicular cell subpopulations. Sperm PUFA are highly susceptible to the oxidative process, due to the high degree of unsaturation. Such oxidative damage is triggered by the insufficient protection exerted by antioxidants, which are mainly enclosed in seminal plasma [11]. Agarwal et al. [12] reported that 20–88% of subfertile men had a high presence of reactive oxygen species (ROS) in the semen.

*n*‐6 PUFA derivatives resulting from arachidonic acid (ARA, 20 : 4*n*‐6) have prothrombotic and proaggregatory properties, whereas *n*‐3 metabolites resulting from eicosapentaenoic acid (EPA, 20 : 5*n*‐3) and docosahexaenoic acid (DHA, 22 : 6*n*‐3) have anti-inflammatory, antiproliferative, and antiatherosclerotic activities [13, 14]. In this view, isoprostanes have recently been identified as markers of *in vivo* and *ex vivo* oxidative damage [15]. Considering that isoprostanes are formed by the esterification of membrane phospholipids, the particular lipid composition of the sperm cell membrane may be one preferential source of their production. Higher levels of F_2_-isoprostanes (F_2_-IsoPs) originating from ARA have been detected in the semen of infertile patients with varicocele than in control and idiopathic infertile men [16] indicating that F_2_-IsoPs could be considered a marker of testis inflammation. Moreover, F_2_-IsoP synthesis appears to be affected by PUFA dietary intake [17] and exogenous administration of *n*‐3 PUFA has been shown to reduce F_2_-IsoPs in patients with Rett syndrome [18]. Esmaeili et al. [19] stated that a high intake of *n*‐3 PUFA enhances sperm characteristics in a dose/time-dependent manner. Furthermore, Martinez-Soto et al. [20] showed that the *n*‐6/*n*‐3 PUFA ratio was lower in sperm of fertile men than in infertile patients. On the other hand, the *n*‐6/*n*‐3 PUFA ratio could be associated with the production of a different class of isoprostanes, namely F_4_-neuroprostanes (F_4_-NeuroPs), in that they are nonenzymatic oxidised products from DHA [21].

In humans, diet is difficult to standardise, thus many researchers use an *in vitro* approach, which evaluates the effect on isolated cells or tissue and does not consider the effect on spermatogenesis. The rabbit is an excellent model of reproductive functions because mature sperm can be easily and continuously collected with an artificial vagina in longitudinal studies [22], and it is particularly interesting for studying sperm alterations due to infection and/or inflammation [23, 24].

Accordingly, this paper aims to study the effect of different *n*‐3 PUFA dietary sources on semen quality, using rabbit buck as the animal model. Spermatogenesis, semen parameters, and the lipid profile have been investigated. One diet was enriched with flaxseed, which has a very high ALA content, whereas fish oil diet directly supplies ALA derivatives (EPA, DPA*n*‐3, and DHA).

## 2. Materials and Methods

### 2.1. Animals and Experimental Design

Thirty New Zealand White rabbit bucks aged 140 days were trained for semen collection for about 30 days. During the training period, the libido (defined as the time between the introduction of a female rabbit and ejaculation) and several sperm traits (volume, concentration, motility rate, and vitality) of each rabbit buck were recorded in order to form homogenous groups.

Fifteen rabbits were selected and divided into three experimental groups (*n* = 5/group) and fed different diets (Table 1):

- The control group was fed ad libitum with the standard diet
- The FLAX group was fed a standard diet, which was supplemented with 10% of extruded flaxseed
- The FISH group was fed a standard diet, which contained 3.5% of fish oil (Nordic Naturals Omega-3®)

**Figure 1:**
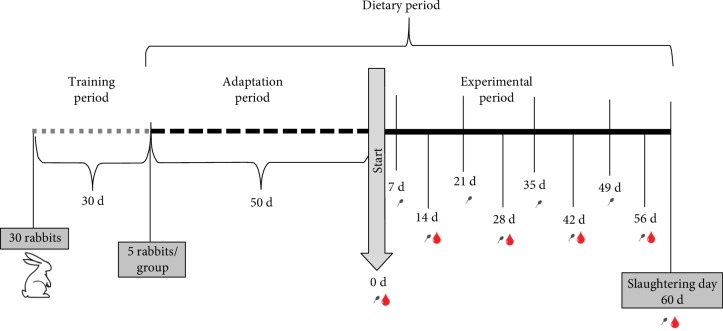
Experimental design of the trial. The gray line shows the training period during which the animals were trained for semen collection, and the seminal traits were analysed in order to create three homogeneous groups. The dashed black line shows the adaptation period during which the animals were fed with three different diets. The solid black line shows the experimental period during which the semen and blood samples were collected, and several traits were analysed.

In Figure 1, the experimental design is reported. The dietary protocol involved 50 days of adaptation during which the rabbits were only monitored for semen collection and a subsequent 60 days (a full spermatogenic cycle) during which sperm determinations were evaluated.

This study was conducted in accordance with the Guiding Principles in the Use of Animals and approved by the Animal Ethics Monitoring Committee of the University of Siena (CEL AOUS; authorization no. 265/2018-PR, ISOPRO 7DF19.23).

### 2.2. Sampling of Semen, Blood, and Reproductive Organs

Semen samples were collected weekly from each rabbit buck (Figure 1), one week before the start of the trial and during the experimental period, producing a total of ten collections.

Semen samples were collected by means of an artificial vagina heated to 38°C with water and immediately transferred to the laboratory [25]. The evaluations of sperm quality were immediately performed on raw samples, as described later. The semen samples were centrifuged at 200 × g for 15 mins; seminal fluid was recovered for the determination of F_2_-IsoPs and F_4_-NeuroPs (butylated hydroxytoluene (BHT) was added at a final concentration of 90 *μ*M). The sperm cells were divided into three aliquots. One aliquot was processed for immunocytochemistry, and the other two aliquots of 10^8^ spermatozoa/mL were stored at -80°C for the evaluation of the oxidative status and FA profile.

Every two weeks, blood samples (2 mL) were taken from the auricular marginal vein using a 2.5 mL syringe fitted with a butterfly needle, after the local application of an anaesthetic cream (EMLA®). Serum was obtained from blood samples coagulated at room temperature for 2 hrs, and then the collection tubes were rimmed and refrigerated at 4°C for 24 hrs before analysis. Plasma was obtained from blood samples collected in tubes containing Na_2_-EDTA and immediately centrifuged at 2,500 × g for 15 mins at 4°C. For the determination of plasma F_2_-IsoPs and F_4_-NeuroPs, BHT was added (90 *μ*M, final concentration).

At the end of the trial (110 days), the rabbits were killed and their testes were accurately removed; a part was fixed for immunocytochemistry, and another part was sampled in sterile tubes and stored at -80°C for the evaluation of the FA profile.

### 2.3. Sperm Quality Assessment

After collection, semen was immediately subjected to analyses to determine the following sperm traits:

- Volume (mL), which was determined by graduated tubes
- Sperm concentration (number of sperm × 10^6^/mL), which was measured by means of a Thoma-Zeiss cell counting chamber with a 40x objective
- Kinetic characteristics, which were analysed by a Computer-Assisted Semen Analyzer (model ISAS®4.0, Valencia, Spain) after appropriate dilution (1/20) with a modified Tyrode's albumin lactate pyruvate buffer [26] at pH 7.4 and 296 mOsm/kg. This system consisted of a negative phase-contrast optic system (Olympus CH-2) equipped with a CCD Sony camera. The set-up parameters were previously established, and the acquisition rate was set at 100 Hz [27]. For each sample, two drops and six microscopic fields were analysed for a total of 300 spermatozoa. Numerous sperm motion parameters were recorded, but only the motility rate (percentage of motile sperm/total sperm) and track speed (*μ*m/s, the sum of the incremental distances moved by the sperm in each frame along the sampled path divided by time) were reported

### 2.4. Oxidative Status of Seminal Plasma and Blood Plasma

The extent of sperm lipid peroxidation (thiobarbituric reactive substances (TBARs) was assessed by measuring malondialdehyde (MDA) along with other substances that are reactive to 2-thiobarbituric acid (TBA), as reported by Mourvaki et al. [26]. The molar extinction coefficient of MDA was 1.56 × 10^5^ 1/M∗cm. The results were expressed as nmol MDA/mL.

Lipid peroxidation was evaluated in the plasma using a spectrophotometer (set at 532 nm, Shimadzu Corporation UV-2550, Kyoto, Japan), which measured the absorbance of TBARs and a 1,1,3,3-tetraethoxypropane calibration curve in sodium acetate buffer (pH = 3.5) [28]. The results were expressed as nmol of MDA/mL of plasma.

### 2.5. Determination of the Levels of Free F_2_-IsoPs and F_4_-NeuroPs

The levels of free F_2_-IsoPs and F_4_-NeuroPs were determined by gas chromatography/negative-ion chemical ionisation tandem mass spectrometry (GC/NICI-MS/MS).

After thawing, the plasma and seminal samples were treated with a volume of acidified water (pH 3) and spiked with a tetradeuterated derivative of prostaglandin F_2*α*_ (PGF_2*α*_-d_4_; 500 pg), as internal standard. Subsequently, solid phase extraction procedures were carried out according to a previously reported methodology [29]. Briefly, each sample (plasma or seminal plasma) was applied to an octadecylsilane (C_18_) cartridge and the eluate was transferred to an aminopropyl (NH_2_) cartridge to collect isoprostanes. All the final eluates were derivatised to convert the carboxyl group of the F_2_-IsoPs or PGF_2*α*_-d_4_ into pentafluorobenzyl ester and the hydroxyl group into trimethylsilyl ethers, as previously reported [29]. The derivatised F_2_-IsoPs and PGF_2*α*_-d_4_ were analysed by GC/NICI-MS/MS. The ions that were determined were the product ions at *m*/*z* 299 and *m*/*z* 303, derived from the [M-181]^−^ precursor ions of 8-iso-PGF_2*α*_, also referred to as 15-F_2t_-IsoP (*m*/*z* 569) and PGF_2*α*_-d_4_ (*m*/*z* 573), respectively [30].

With reference to F_4_-NeuroPs, the mass ions that were determined were the product ions at *m*/*z* 323 and *m*/*z*303, derived from the [M-181]^−^ precursor ions of 10-F_4t_NeuroPs, considered as the most represented F_4_-NeuroPs (*m*/*z* 593) [21] and PGF_2*α*_-d_4_ (*m*/*z* 573), respectively.

### 2.6. Fatty Acid Profiles of the Sperm and Testis

The lipid extraction from the raw semen and testis was performed according to the method of Folch et al. [31], and the esterification was carried out following the procedure of Christie [32]. The transmethylation procedure was conducted using eicosenoic acid methyl esters (Sigma-Aldrich) as internal standard. The recovery rates of the internal standard were 89 ± 4% and 83 ± 3% in the semen and the testis, respectively.

The FA composition was determined using a Varian gas-chromatograph (CP-3800) equipped with a flame ionisation detector and a capillary column of 100 m length × 0.25 mm × 0.2 *μ*m film (Supelco, Bellefonte, PA, USA). Helium was used as the carrier gas with a flow of 0.6 mL/min. The split ratio was 1 : 20. The oven temperature was programmed as reported by Mattioli et al. [33]. Individual FAME were identified by comparing the relative retention times of peaks in the sample with those of a standard mixture (FAME Mix Supelco; 4 : 0 to 24 : 0) plus *cis*-9 *cis*-12 C18 : 2; *cis*-9 *cis*-12 *cis*-15 C18 : 3; and *cis*-9 *cis*-12 *cis*-15 C18 : 3 (all from Sigma-Aldrich). The FA were expressed as % of total FA. The average amount of each FA was used to calculate the sum of the total saturated fatty acid (SFA), monounsaturated fatty acid (MUFA), and PUFA.

To evaluate the efficiency of the metabolising precursors (LA and ALA) into LC PUFA, the ratio LC PUFA/precursors was calculated for both *n*‐3 and *n*‐6 PUFA [34].

### 2.7. Testosterone Evaluation in Blood Plasma

The testosterone concentration in the rabbit serum was performed by radioimmunoassay assay (RIA). The kit that was used was the Testosterone (^125^I) RIA KIT (Ref: RK-61M Institute of Isotopes Co. Ltd., Bucharest). This assay is based on the competition between unlabelled testosterone and a fixed quantity of ^125^I-labelled testosterone for a limited number of binding sites on a testosterone-specific antibody. This allows the reaction of a fixed amount of tracer and antibody with different amounts of unlabelled ligand, the amount of tracer bound by the antibody being inversely proportional to the concentration of unlabelled ligand. Upon the addition of a magnetisable immunosorbent, the antigen-antibody complex is bound on solid particles, which are then separated by either magnetic sedimentation or centrifugation. Counting the radioactivity of the solid phase enables the construction of a standard curve and samples to be quantitated. This method showed the following cross-reactions: 100% testosterone, 35% 5*α*-dihydrotestosterone, 0.8 5*β*-dihydrotestosterone, 0.01 17*β*-estradiol, and 0.01 cortisol. The percentage of interference was calculated using the formula of Abraham: *X*/*Y*∗100, where *X* and *Y* were, respectively, the weight of the substance to be determined and the weight of the interfering substance, so as to reduce the binding capacity by 50%. The sensitivity was the lowest dose of testosterone that was 5% lower than the initial binding capacity.

### 2.8. Immunohistochemical Analysis

#### 2.8.1. Testicular Tissue

The testes of the rabbit bucks that were fed the control and *n*‐3-enriched diets were cut into small blocks and treated with 10% buffered formalin for 24 hrs at 4°C and were then washed in water for 1 h. After fixation, the tissues were dehydrated in a series of ethanol (50%, 75%, 95%, and 100%) and cleared with xylene. The specimens were treated with three infiltrations of molten paraffin at 60°C for 1 h and then solidified at room temperature. The obtained blocks were sectioned using a Leica RM2125 RTS microtome (Leica Biosystem, Germany); sections (4 *μ*m) were collected in glass slides and stained using the hematoxylin-eosin method for routine histology. The paraffin sections from the testicular tissue of the control and treated rabbits were deparaffinised with xylene and then treated in a series of ethanol concentrations (100%, 90%, 80%, and 70%) for 5 mins and, finally, in water to rehydrate the tissue. For antigen retrieval, the sections were washed and treated with heat-induced epitope retrieval 1 (HIER 1) buffer (10 mM sodium citrate) at pH 6 for 20 mins at 95°C. Specimens were treated overnight at 4°C with rabbit anti-DHA and FITC-linked rabbit anti-EPA polyclonal antibodies (MyBioSource Inc., San Diego, CA, USA) at a dilution of 1 : 40.

After three washes for 10 mins in phosphate-buffered saline (PBS), the slides treated with anti-DHA or EPA and were incubated with goat anti-rabbit FITC-conjugated antibody (Sigma-Aldrich, Milan, Italy), diluted 1 : 100 for 1 h at room temperature. The slides were washed with PBS three times and mounted with 1,4-diazabicyclo(2.2.2)octane (DABCO, Sigma-Aldrich, Milan, Italy).

#### 2.8.2. Ejaculate Sperm

Briefly, washed, smeared sperm [35] were incubated with the same polyclonal antibodies (DHA and EPA).

All the samples were observed under a Leica DMI6000 microscope (Leica Microsystems, Germany) with a 63x objective, and the images were acquired using a Leica AF6500 Integrated System for Imaging and Analysis (Leica Microsystems, Germany). In detail, the images were obtained with HCX PL FLUOTAR 63x/1.25 oil objective; filters for TRIC and FITC were selected. The micrographs were not modified with image elaboration software.

The specificity of the antibodies, guaranteed in the datasheets of both antibodies, was also evaluated by omitting the primary antibody.

### 2.9. Statistical Evaluations

All the traits (semen volume, concentration, kinetics, oxidative traits, testosterone, and isoprostanes) had repeated values and were analysed with a mixed model to evaluate the fixed effect of diet (control, flaxseed, and fish oil) [36] and the random effect of rabbit buck over time. LSmeans and pooled SE were reported. The Bonferroni correction was applied for multiple comparisons. The significance was set at *P* < 0.05.

## 3. Results

The FA profile of *n*‐3 supplemented diets (Table 2) was richer in *n*‐3 (46.00 and 26.40% vs. 11.35% in FLAX, FISH, and control, respectively) and lower in *n*‐6 (mainly LA) compared with that in the control. The *n*‐6/*n*‐3 ratio was about the same in both the *n*‐3 supplemented groups (0.50 and 0.80 in FLAX and FISH, respectively) and much lower than that in the control (4.53).

The TBARs of blood plasma were not significantly (*P* > 0.05) increased by dietary *n*‐3 (Table 3). On the contrary, seminal plasma had about three times more TBARs in the groups fed supplemental *n*‐3.

Conversely, F_2_-IsoPs in the blood plasma of *n*‐3-enriched diets decreased by about 28% and 38%, respectively, in FLAX and FISH groups (Table 4) and the seminal plasma had the same tendency (-39% and -35%). On the contrary, the levels of F_4_-NeuroPs both in blood and in seminal plasma were double the levels observed in the control group.

The sperm kinetic traits (motility rate and track speed) significantly improved in bucks fed with *n*‐3 sources (Table 5).

The *n*‐3 PUFA source also affected the FA profile of the testes (Table 6) and the sperm (Table 7), increasing the DHA in the sperm from the FLAX and FISH groups by 10 and 30 times, respectively, in comparison with the control group. Almost the same tendency was shown in testes with a concomitant reduction of *n*‐6 LC PUFA (mainly DPA*n*‐6, 22:5*n*‐6) when dietary *n*‐3 was added.

In Table 8, the metabolic indexes of the testes and sperm of the different dietary groups are compared. These indexes (e.g., *n*‐6 LC/LA and *n*‐3 LC/ALA) roughly estimate the anabolic ability of the precursors (LA and ALA) to produce long-chain derivatives (ARA, DPA*n*‐6, EPA, DPA*n*‐3, and DHA). All these indexes were affected by dietary PUFA; in particular, the testes of *n*‐3-enriched groups showed lower *n*‐6 LC PUFA production and higher *n*‐3 LC PUFA, with respect to the control.

**Figure 2:**
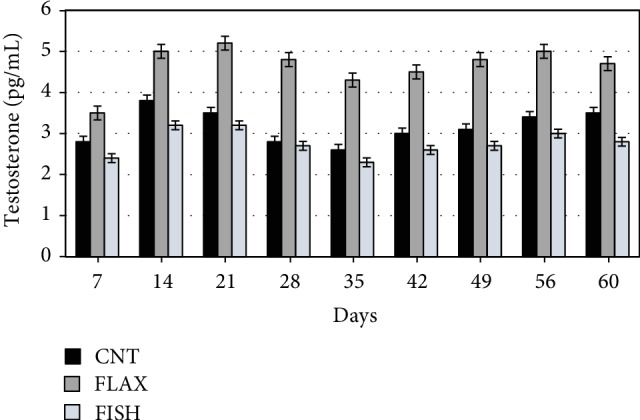
Effect of experimental diets on the testosterone (pg/mL) concentration in blood. The black bar shows the control (C), the dark gray bar shows the FLAX group, and the light gray bar shows the FISH group.

Figure 2 shows the blood testosterone trend, which was significantly affected by time (age of bucks) and dietary groups. The effect of time, which is common in all the groups, is probably related to the maturation of the animals.

Regarding the effect of diet only, the FLAX group showed higher testosterone concentration with respect to the others. The group fed with fish oil showed slight differences over time and was almost similar to the control.

**Figure 3:**
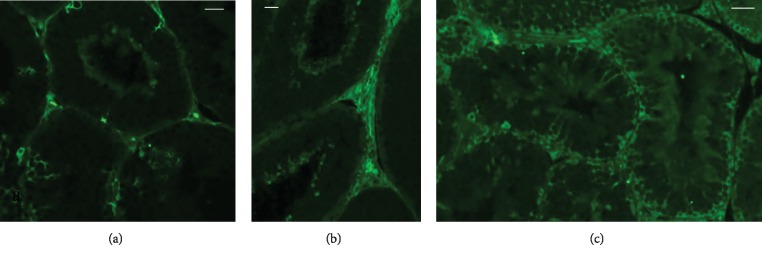
Immunolocalisation of DHA in testicular tissue from rabbit bucks fed control (a) and *n*‐3 enriched diets ((b) FLAX; (c) FISH). A faint fluorescent stain in the germ cells and interstitial tissue (Leydig cells) is shown in (a). A high-labelled intensity in the interstitial tissue and germ cells is evident in (b) and (c). In (c), the signal is also present in the Sertoli cells. Bars: 30 *μ*m (a); 20 *μ*m (b); 40 *μ*m (c).

Immunofluorescent staining was performed on the paraffin-embedded testis tissue of the rabbit bucks fed control or *n*‐3-enriched diets. As shown in Figure 3, we observed that DHA was strongly expressed in the tubules and the interstitial tissue of testis from the *n*‐3-enriched groups (Figures 3(b) and 3(c)) compared with the control (Figure 3(a)).

**Figure 4:**
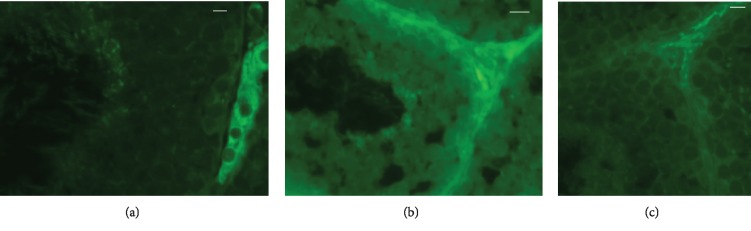
Immunolocalisation of EPA in testicular tissue from rabbit bucks fed control (a) and *n*‐3 enriched diets ((b) FLAX; (c) FISH). (a) EPA labelling is evident in interstitial tissue, and a faint fluorescent stain in germ cells is also shown in the control testis. A high-labelled intensity in the interstitial tissue and in germ cells is evident in FLAX (b) and FISH (c). Bars: 15 *μ*m (a and c); 10 *μ*m (b).

The labelling of EPA (Figures 4(a)–4(c)) was evident in interstitial tissue; it was weak in the seminiferous tubules of the control rabbits (Figure 4(a)), whereas it was very bright (FLAX, Figure 4(b); FISH, Figure 4(c)) in all stages of the spermatogenic process of the rabbit bucks fed *n*‐3 enriched diets.

**Figure 5:**
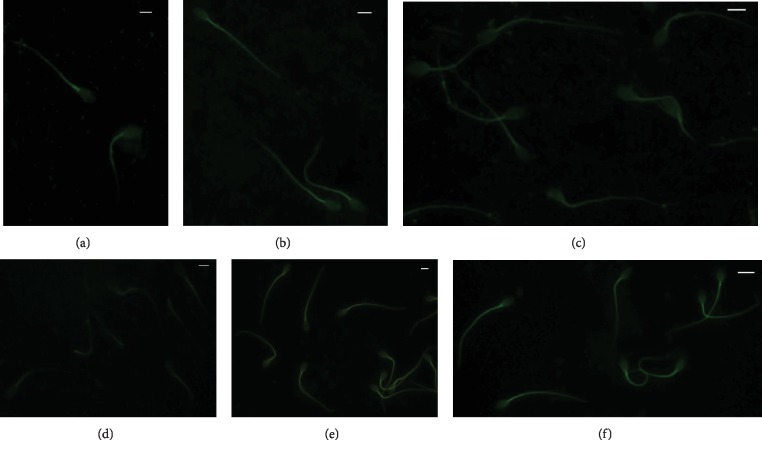
Spermatozoa from rabbit bucks fed control (a, d) and *n*‐3 enriched diets ((b and e) FLAX; (c and f) FISH) treated with anti-DHA (a–c) and EPA (d–f) antibodies. A fluorescent stain was evident at the midpiece level in the control (a, d), and a high-labelled intensity stain along the sperm tail and, frequently, in the basal portion of the head was shown in the sperm from the treated groups ((b and e) FLAX; (c and f) FISH). Bars: 4 *μ*m (a and b); 5 *μ*m (c, d, and e); 6 *μ*m (f).

Ejaculated sperm from rabbit bucks fed control and *n*‐3 enriched diets (FLAX and FISH) were treated with anti-DHA and EPA antibodies. In the sperm from the control rabbits, both DHA and EPA showed a weak fluorescent staining localised in the middle piece of the tail (Figures 5(a) and 5(d)); in the sperm from the treated groups, the fluorescent staining was strongly evident in the entire tail and often in the postacrosome region (FLAX, Figures 5(b) and 5(e); FISH, Figures 5(c) and 5(f)).

## 4. Discussion

As expected, the FA profile of the testes and sperm was affected by both dietary *n*‐3 supplementations. Feeding male rabbits with fish oil resulted in the accumulation of *n*‐3 LC PUFA in the testes and sperm. Flaxseed administration mainly increased ALA, but it also confirmed a certain LC PUFA synthesis and the ability of animals (liver and testis) to elongate and desaturate ALA [26, 37]. In agreement with our previous research [33], we demonstrated the ability of the reproductive tissues (e.g., ovary) to efficiently synthesize and accumulate *n*‐3 LC PUFA. Similarly, in pig testes an increase in *n*‐3 LC PUFA content (eleven-fold and two-fold more for EPA and DHA, respectively) was reported with dietary tuna oil administration [38], partially due to the higher fatty acid 2 (FADS2) gene expression.

The LC PUFA deposition in the sperm membrane probably contributes to the improvement of membrane fluidity and the relative motion traits of cells. Indeed, the track speed of the *n*‐3 PUFA groups increased by about 30%. As a result, the tracing of dietary PUFA in testes and sperm is particularly interesting. Both diet supplementations may be able to increase DHA and EPA in two ways: being directly provided by fish oil or by flax, which provides their precursor.

An increased presence of DHA and EPA in the testes was also confirmed using the immunofluorescence technique. The DHA signal appeared to have increased in the interstitial tissue, putatively in the Leydig cells, germ cells, and Sertoli cells of rabbits belonging to both *n*‐3 supplemented groups. In the testis, phospholipid DHA promotes sperm membrane structural changes that are required for a regular spermatogenesis [39]. Moreover, the presence of certain FA in Sertoli cells positively influences spermatogenesis for the significant role played by Sertoli cells during sperm maturation [40].

In a recent *in vitro* study, *n*‐3 PUFA have been classified as protective for Sertoli cells [41]. EPA was mainly localised in the interstitial tissue of the control rabbits, but it was more abundant in the testes from the *n*‐3 enriched groups, both in the interstitial tissue and germ cells. It is known that Leydig cells, producing testosterone, play a major role in spermatogenesis, and the enrichment of LC PUFA in the interstitial tissue of testes in *n*‐3 PUFA groups may indicate a good status of Leydig cells. However, the secretory activity (e.g., testosterone) of these cells was affected in a different way by the *n*‐3 dietary sources. Flaxseed, being one of the richest sources of phytoestrogens (lignans), which act as hormone-like compounds, may increase sex-hormone-binding-globulin synthesis by consequently stimulating testosterone production [42]. Qi et al. [43] have suggested that dietary flaxseed improves semen quality by increasing the testosterone hormone secretion, which may be related to higher StAR and P450scc mRNA and SF-1 expression. Moreover, Li et al. [44] showed that dietary linseed oil supplemented during peripuberty stimulates steroidogenesis and testis development in rams.

On the other hand, our results agree with Castellano et al. [38], who reported that the dietary supplementation of EPA and DHA partly reduced steroidogenesis. This may be due to an inhibition of prostaglandin release from ARA [38] and/or a reduction of the gene expression implicated in steroidogenic pathways [45].

Furthermore, the sperm from the control rabbits showed the EPA and DHA were localised in the sperm midpiece, whereas in the sperm from the *n*‐3 supplemented groups, these FA increased and were distributed throughout the entire sperm tail. This localisation may be associated with an improvement in the sperm plasma membrane fluidity and an increase of the kinetic traits [46]. Mourvaki et al. [26] showed that dietary DHA in rabbit sperm is mainly incorporated into the midpiece and this enhancement is positively associated with sperm movement. In humans, other researchers [47] have reported that a higher intake of *n*‐3 PUFA was positively correlated with sperm morphology, total sperm count, and sperm cell density, and that EPA has a positive influence on eel sperm performance [48]. Our data indicate that the membrane of the sperm head partly incorporates EPA and DHA, and this enrichment may influence two fundamental steps in the fertilisation process, such as acrosome reaction and membrane fusion [49]. It is known that changes in the sperm FA profile, essential for fertilisation, occur during cauda epididymal maturation [50]; however, this aspect has been not investigated here.

As already stated, PUFA are very susceptible to free radical attack, and oxidation increases as the number of double bonds increases. Therefore, the oxidisability of PUFA is correlated with the number of methylene groups located between two bonds and increases approximately two-fold for each additional methylene group [51].

Accordingly, although the diets had a high level of antioxidants (200 mg/kg vitamin E vs. the standard recommendation of 50 mg/kg) [52, 53], the increase of PUFA in tissues and cells was accompanied by a reduction in the oxidative stability. In the present study, the extent of the TBARs was assessed by the MDA levels; it is conceivable that the supplementation of *n*‐3 PUFA, increasing the substrate, directly influences the amount of MDA. It should be emphasised that the seminal plasma appeared more susceptible to peroxidation than the blood. Tissues exhibit different susceptibility to oxidative stress; the male reproductive system, similar to the central nervous system, may be particularly vulnerable to oxidative damage due to the limited efficiency of the antioxidant system and abundant PUFA content that is highly susceptible to lipid peroxidation [54].

At the same time, the isoprostanes (F_2_-IsoPs and F_4_-NeuroPs) did not appear to be strictly correlated with the overall oxidative status in both the blood and the semen. In particular, F_2_-IsoPs, which are considered the “gold standard” for measuring oxidative stress in the whole body [55], were higher in the control group and the F_4_-NeuroPs were lower when compared with the groups supplemented with *n*‐3 PUFA. F_2_-IsoPs decreased by up to 40% suggesting that *n*‐3 effectively decreases the proinflammatory isoprostanes derived from arachidonate. At the same time, F_4_-NeuroPs, deriving from DHA, increased by almost the same amount.

This investigation suggests that the F_2_-IsoP level should be interpreted with caution as a marker of peroxidation in *n*‐3 rich tissues (e.g., the reproductive system and the nervous system) or when that is induced by dietary *n*‐3 PUFA. In such circumstances, it cannot be concluded that low F_2_-IsoPs indicate no oxidative stress, as the reduction of F_2_-IsoPs after the supplementation of *n*‐3 PUFA might occur through several nonenzyme unidentified pathways and be accompanied by a comparable increase of F_4_-NeuroP. Such an increase in neuroprostanes could be related to the peculiar localisation of the F_4_-NeuroP precursor fatty acid (i.e., DHA). DHA, unlike ARA, is widely concentrated in the brain and testis, with relatively low levels in other organs [56]. Thus, in neural tissues, F_4_-NeuroPs are the most abundant products of an oxidative nonenzyme pathway [57] and their quantification provides a highly selective index for *in vivo* neuronal oxidative damage.

To our knowledge, no other research has shown that F_4_-NeuroPs in seminal plasma are affected by diet and were at low levels in animals fed with control diets (high LA and low *n*‐3 PUFA). When additional *n*‐3 PUFA are furnished, in *n*‐3 rich tissues, these molecules increased and could act as specific biomarkers of *n*‐3 peroxidation [58, 59]. Furthermore, other molecules resulting from the nonenzyme oxidation of PUFA could be also generated: i.e., F_3_-IsoPs from EPA and E_1_-IsoPs from ALA. In this regard, Roberts and Milne [60] found that with *n*‐3 dietary supplementation, the brain level of IsoPs (F_3_-IsoPs) produced from the oxidation of EPA significantly exceeds that of the F_2_-IsoPs generated from ARA because EPA is more easily oxidisable (one more double bond than ARA).

Gao et al. [61] confirm that *n*‐3 PUFA supplementation decreased F_2_-IsoPs (up to 64%) in the heart tissue and led to the formation of F_3_-isoprostanes, proving that *n*‐3 effectively decreases levels of proinflammatory F_2_-IsoPs formed from arachidonate. Such an observation is crucial because F_2_-IsoPs are generally considered as proinflammatory molecules associated with the pathophysiological effect of oxidant stress. It is thus interesting to realise that the mechanism by which *n*‐3 LC PUFA prevents certain diseases resides in its ability to decrease F_2_-IsoP generation [60].

Syta-Krzyzanowska et al. [62] showed that in cerebral tissues, where the level of DHA is high (similarly to testes), a pathological situation (e.g., aneurysm) leads to the enhancement of lipid peroxidation with an increase in plasma F_2_-IsoPs and F_4_-NeuroPs (more than three-fold and eleven-fold, respectively). Related work [63] suggested that the metabolism of LC PUFA yields oxidised bioactive compounds that mediate its effects. Serhan and Chiang [63] described novel anti-inflammatory hydroxylated EPA metabolites (termed E-series resolvins) deriving from the enzymatic-mediated oxidation of EPA. These findings have led to considerable interest in determining other oxidation products of EPA that may mediate the anti-inflammatory effects of this FA. It is conceivable that the increased sperm motility could be related to an increase of proresolving mediators (derived from DHA and EPA) or the biological activity of nonenzymatic *n*‐3 PUFA metabolites (i.e., F_4_-NeuroPs). However, the role and effect of these classes of compounds are still not entirely known.

## 5. Conclusions

The dietary administration of *n*‐3 PUFA resulted in an enrichment of DHA and EPA in rabbit sperm and testes and indicated that the rabbit is a suitable model for the study of the spermatogenic process. Clearly, the fish oil diet supplemented with LC PUFA influenced the LC PUFA composition of testes and sperm more than the flax diet, which furnished the precursor. The *n*‐3 supplementation improved the sperm motility rate and track speed in male rabbits; however, such an increased amount of PUFA negatively affected the sperm oxidative status, which could not be revealed with F_2_-IsoP evaluations, because it is mainly derived from ARA (*n*‐6 PUFA) metabolism. Therefore, F_2_-IsoPs cannot be considered a good marker of oxidation when a diet rich in *n*‐3 PUFA is provided. Furthermore, such oxidative thrust was mainly generated in the tissues with a higher concentration of *n*‐3 PUFA, as demonstrated by the increased amount of F_4_-NeuroPs. Further studies are in progress to test the effect of *n*‐3 dietary supplementation on other rabbit tissues that are mainly involved in the *n*‐3 PUFA-metabolic pathways (liver and brain).

## Figures and Tables

**Table 1:** Formulation and proximate analysis of the control and *n*‐3-enriched diets.

Ingredients (g/kg)	Control	FLAX	FISH
Dehydrated alfalfa meal	300	380	380
Soybean meal 44%	150	100	150
Barley meal	410	310	335
Wheat bran	52	52	52
Soybean oil	30	—	—
Extruded flaxseed	—	100	—
Fish oil^∗^	—	—	35
Beet molasses	20	10	10
Calcium carbonate	7	7	7
Calcium diphosphate	13.5	13.5	13.5
Salt	7	7	7
DL-methionine	0.5	0.5	0.5
Vitamin-mineral premix^†^	10	10	10
Crude protein	175	174	175
Ether extract	480	472	425
Crude Fiber	124	137	130
Ash	89	84	90

^∗^Nordic Naturals Omega-3®=purified deep sea fish oil (from anchovies and sardines) containing EPA—330 mg/100 g, DHA—220 mg/100 g, and other *n*‐3 LC PUFA—140 mg/100 g+*α*-tocopherol for preservation. ^†^Per kg diet: vitamin A—11.000 IU; vitamin D_3_—2000 IU; vitamin B_1_—2.5 mg; vitamin B_2_—4 mg; vitamin B_6_—1.25 mg; vitamin B_12_—0.01 mg; alpha-tocopheryl acetate—200 mg; biotine—0.06 mg; vitamin K—2.5 mg; niacin—15 mg; folic acid—0.30 mg; D-pantothenic acid—10 mg; choline—600 mg; Mn—60 mg; Fe—50 mg; Zn—15 mg; I—0.5 mg; Co—0.5 mg.

**Table 2:** FA profile of the control and *n*‐3-enriched diets.

	Control (% of total FA)	FLAX (% of total FA)	FISH (% of total FA)	Pooled SE
SFA	19.80^a^	15.40^a^	38.10^b^	1.82
MUFA	17.40	15.80	14.50	0.87
PUFA	62.80^a^	68.80^a^	47.40^b^	5.12
LA	50.45^b^	22.30^a^	20.50^a^	2.11
ALA	11.15^a^	45.80^b^	18.50^a^	1.42
LC PUFA*n*‐3	—	—	10.50	1.00
EPA	—	—	3.50	0.21
DHA	—	—	4.20	0.28
*n*‐6	51.45^b^	22.80^a^	21.00^a^	2.35
*n*‐3	11.35^a^	46.00^c^	26.40^b^	1.55
*n*‐6/*n*‐3	4.53^b^	0.50^a^	0.80^a^	0.01

a, b, and c on the same line means *P* ≤ 0.05. Legend: SFA—saturated fatty acids; MUFA—monounsaturated fatty acids; PUFA—polyunsaturated fatty acids; LA—linoleic acid; ALA—*α*-linolenic acid; LC PUFA—long-chain PUFA; EPA—eicosapentaenoic acid; DHA—docosahexaenoic acid.

**Table 3:** TBARs in the blood and seminal plasma of rabbit bucks fed the control or *n*‐3-enriched diets.

	Blood plasma (nmol MDA/mL)	Seminal plasma (nmol MDA/mL)
Control	42.12	3.38^a^
FLAX	48.74	10.66^b^
FISH	45.59	11.66^b^
Pooled SE	3.55	0.87

a and b on the same line column means *P* ≤ 0.05.

**Table 4:** Isoprostanes (F_2_-IsoPs) and neuroprostanes (F_4_-NeuroPs) in the blood and seminal plasma of the rabbit bucks fed the control or *n*‐3-enriched diets.

	F_2_-IsoPs (pg/mL)		F_4_-NeuroPs (pg/mL)	
	Blood plasma	Seminal plasma	Blood plasma	Seminal plasma
Control	150.21^b^	124.08^b^	8.52^a^	14.54^a^
FLAX	108.75^a^	75.78^a^	16.85^b^	25.04^b^
FISH	92.28^a^	80.01^a^	17.77^b^	27.51^b^
Pooled SE	8.05	7.22	1.45	1.83

a and b on the same column means *P* ≤ 0.05.

**Table 5:** Seminal traits of the rabbit bucks fed control or *n*‐3-enriched diets.

	Control	FLAX	FISH	Pooled SE
Volume (mL)	0.61	0.57	0.55	0.04
Sperm concentration (10^6^/mL)	255.31	280.10	248.32	18.53
Motility rate (%)	63.03^a^	77.22^b^	77.55^b^	5.40
Track speed (*μ*m/sec)	180.91^a^	239.94^b^	228.72^b^	15.54

a and b on the same line means *P* ≤ 0.05.

**Table 6:** Testes FA profile of the rabbit bucks fed the control or *n*‐3-enriched diets.

	% of total evaluated FA	Control	FLAX	FISH	Pooled SE
Saturated fatty acid (SFA)	Miristic (C14 : 0)	2.50	2.80	1.80	1.35
	Palmitic (C16 : 0)	25.78	26.19	26.81	2.02
	Stearic (C18 : 0)	11.14	10.00	10.27	1.91
	SFA	39.43	38.99	38.88	2.90

Monounsaturated fatty acid (MUFA)	Palmitoleic (C16 : 1)	2.58	2.94	4.47	0.22
	Oleic (C18 : 1)	16.36	17.89	19.49	1.55
	MUFA	18.95	20.83	23.97	1.85

*n*‐6 polyunsaturated acids (PUFA)	Linoleic (LA) (C18 : 2*n*‐6)	11.85	13.37	14.01	2.01
	Eicosatrienoic (C20 : 3*n*‐6)	5.87	5.39	3.67	0.08
	Arachidonic (ARA) (C20 : 4*n*‐6)	8.46	5.87	6.27	0.04
	Adrenic (C22 : 4*n*‐6)	2.32	1.60	1.18	0.24
	All-*cis* 4,7,10,13,16-docosapentaenoic (DPA*n*‐6) (C22 : 5*n*‐6)	10.04^b^	5.88^a^	7.01^ab^	3.34
	*n*‐6 PUFA	38.53^b^	32.11^a^	32.15^a^	0.22

*n*‐3 polyunsaturated acids (PUFA)	*α*-Linolenic (ALA) (C18 : 3*n*‐3)	2.03^a^	7.02^b^	3.00^a^	0.05
	Eicosapentaenoic (EPA) (C20 : 5*n*‐3)	0.01	0.05	0.04	0.09
	All-*cis*-7,10,13,16,19-docosapentaenoic (DPA*n*‐3) (C22 : 5*n*‐3)	0.01	0.05	0.09	1.50
	Docosahexaenoic (DHA) (C22 : 6*n*‐3)	0.07^a^	0.83^b^	1.46^b^	0.10
	*n*‐3 PUFA	2.13^a^	7.91^b^	4.58^b^	3.85
	*n*‐6/*n*‐3	18.13^c^	4.06^a^	7.02^b^	25.2

a, b, and c on the same line means *P* ≤ 0.05.

**Table 7:** Sperm FA profile of the rabbit bucks fed the control or *n*‐3-enriched diets.

	% of total evaluated FA	Control	FLAX	FISH	Pooled SE
Saturated fatty acid (SFA)	Miristic (C14 : 0)	2.70^a^	4.00^b^	4.20^b^	1.35
	Palmitic (C16 : 0)	23.10	25.80	24.65	2.03
	Stearic (C18 : 0)	22.90^b^	23.90^b^	17.75^a^	1.90
	SFA	48.70^a^	53.70^b^	46.60^a^	2.89

Monounsaturated fatty acid (MUFA)	Palmitoleic (C16 : 1)	0.85	1.05	0.90	0.11
	Oleic (C18 : 1)	15.75^a^	17.50^ab^	20.50^b^	0.97
	MUFA	16.60^a^	19.55^ab^	21.40^b^	0.99

*n*‐6 polyunsaturated acids (PUFA)	Linoleic (LA) (C18 : 2*n*‐6)	7.15^b^	3.89^a^	4.65^a^	2.10
	Eicosatrienoic (C20 : 3*n*‐6)	0.65^a^	1.72^b^	0.55^a^	0.08
	Arachidonic (ARA) (C20 : 4*n*‐6)	1.55^b^	0.65^a^	0.70^a^	0.04
	Adrenic (C22 : 4*n*‐6)	0.30	0.21	0.31	0.03
	All-*cis*-4,7,10,13,16-docosapentaenoic (DPA*n*‐6) (C22 : 5*n*‐6)	24.20^b^	15.88a	12.80^a^	3.48
	*n*‐6 PUFA	33.85^b^	22.35^ab^	19.01^a^	3.85

*n*‐3 polyunsaturated acids (PUFA)	*α*-Linolenic (ALA) (C18 : 3*n*‐3)	0.20^a^	1.29^c^	0.45^b^	0.10
	Eicosapentaenoic (EPA) (C20 : 5*n*‐3)	0.15^a^	0.45^a^	4.25^b^	0.10
	All-*cis*-7,10,13,16,19-socosapentaenoic (DPA*n*‐3) (C22 : 5*n*‐3)	0.10^a^	0.31^a^	1.65^b^	0.09
	Docosahexaenoic (DHA) (C22 : 6*n*‐3)	0.25^a^	2.25^b^	6.65^c^	1.35
	*n*‐3 PUFA	0.70^a^	4.20^b^	13.00^c^	0.20
	*n*‐6/*n*‐3	48.36^b^	5.32^a^	1.46^a^	3.88

a, b, and c on the same line means *P* ≤ 0.05.

**Table 8:** Metabolic indexes of testes and sperm in the rabbit bucks fed the control or *n*‐3-enriched diets.

	Control	FLAX	FISH	Pooled SE
*Testes*				
*n*‐6 LC PUFA	26.70^b^	18.74^a^	18.14^a^	1.02
*n*‐3 LC PUFA	0.09^a^	0.89^b^	1.58^b^	0.15
*n*‐6 LC/LA	2.25^b^	1.40^a^	1.29^a^	0.11
*n*‐3 LC/ALA	0.04^a^	0.13^ab^	0.53^b^	0.08
*Sperm*				
*n*‐6 LC PUFA	26.78^b^	19.46^a^	14.36^a^	1.15
*n*‐3 LC PUFA	0.50^a^	2.91^b^	12.55^b^	0.38
*n*‐6 LC PUFA/LA	3.75	4.75	3.09	0.27
*n*‐3 LC PUFA/ALA	2.50^a^	2.26^a^	27.89^b^	1.10

a, b, and c on the same line means *P* ≤ 0.05. Legend: ALA—*α*-linolenic acid; LA—linoleic acid; LC PUFA—long-chain PUFA.

## Data Availability

The raw data (fatty acids and isoprostanes) used to support the findings of this study are available from the corresponding author upon request.

## References

[1] Levine H., Jørgensen N., Martino-Andrade A. (2017). Temporal trends in sperm count: a systematic review and meta-regression analysis. *Human Reproduction Update*.

[2] Tavilani H., Doosti M., Nourmohammadi I. (2007). Lipid composition of spermatozoa in normozoospermic and asthenozoospermic males. *Prostaglandins, Leukotrienes and Essential Fatty Acids*.

[3] Van Tran L., Malla B. A., Kumar S., Tyagi A. K. (2017). Polyunsaturated fatty acids in male ruminant reproduction—a review. *Asian-Australasian journal of animal sciences*.

[4] Patterson E., Wall R., Fitzgerald G. F., Ross R. P., Stanton C. (2012). Health implications of high dietary omega-6 polyunsaturated fatty acids. *Journal of nutrition and metabolism*.

[5] Retterstol K., Haugen T. B., Tran T. N., Christophersen B. O. (2001). Studies on the metabolism of essential fatty acids in isolated human testicular cells. *Reproduction*.

[6] Björkgren I., Alvarez L., Blank N. (2016). Targeted inactivation of the mouse epididymal beta-defensin 41 alters sperm flagellar beat pattern and zona pellucida binding. *Molecular and Cellular Endocrinology*.

[7] Koeberle A., Shindou H., Harayama T., Yuki K., Shimiz T. (2012). Polyunsaturated fatty acids are incorporated into maturating male mouse germ cells by lysophosphatidic acid acyltransferase 3. *The FASEB Journal*.

[8] Vallés A. S., Aveldaño M. I., Furland N. E. (2014). Altered lipid homeostasis in Sertoli cells stressed by mild hyperthermia. *PloS one*.

[9] Fasel N., McMillian K., Jakop U. (2018). Modification of sperm fatty acid composition during epididymal maturation in bats. *Reproduction*.

[10] Harbige L. S. (2003). Fatty acids, the immune response, and autoimmunity: a question of *n*‐6 essentiality and the balance between *n*‐6 and *n*‐6. *Lipids*.

[11] Castellini C., Lattaioli P., Dal Bosco A., Minelli A., Mugnai C. (2003). Oxidative status and semen characteristics of rabbit buck as affected by dietary. *Reproduction Nutrition Development*.

[12] Agarwal A., Virk G., Ong C., Du Plessis S. S. (2014). Effect of oxidative stress on male reproduction. *The world journal of men's health*.

[13] Wood J. N. (1990). Essential fatty acids and their metabolites in signal transduction. *Biochemical Society Transactions*.

[14] Serhan C. N., Yacoubian S., Yang R. (2008). Anti-inflammatory and proresolving lipid mediators. *Annual Review of Pathology: Mechanisms of Disease*.

[15] Basu S. (2010). Bioactive eicosanoids: role of prostaglandin F 2*α* and F 2-isoprostanes in inflammation and oxidative stress related pathology. *Molecules and Cells*.

[16] Collodel G., Moretti E., Longini M., Pascarelli N. A., Signorini C. (2018). Increased F2-isoprostane levels in semen and immunolocalization of the 8-iso prostaglandin F2*α* in spermatozoa from infertile patients with varicocele. *Oxidative Medicine and Cellular Longevity*.

[17] Maffei S., De Felice C., Cannarile P. (2014). Effects of *ω*‐3 PUFAs supplementation on myocardial function and oxidative stress markers in typical Rett syndrome. *Mediators of Inflammation*.

[18] De Felice C., Signorini C., Leoncini S. (2012). The role of oxidative stress in Rett syndrome: an overview. *Annals of the New York Academy of Sciences*.

[19] Esmaeili V., Shahverdi A. H., Moghadasian M. H., Alizadeh A. R. (2015). Dietary fatty acids affect semen quality: a review. *Andrology*.

[20] Martínez-Soto J. C., Landeras J., Gadea J. (2013). Spermatozoa and seminal plasma fatty acids as predictors of cryopreservation success. *Andrology*.

[21] Galano J. M., Mas E., Barden A. (2013). Isoprostanes and neuroprostanes: total synthesis, biological activity and biomarkers of oxidative stress in humans. *Prostaglandins and Other Lipid Mediators*.

[22] Castellini C., Mourvaki E., Sartini B. (2009). In vitro toxic effects of metal compounds on kinetic traits and ultrastructure of rabbit spermatozoa. *Reproductive Toxicology*.

[23] Brecchia G., Cardinali R., Mourvaki E. (2010). Short- and long-term effects of lipopolysaccharide-induced inflammation on rabbit sperm quality. *Animal Reproduction Science*.

[24] Brecchia G., Menchetti L., Cardinali R. (2014). Effects of a bacterial lipopolysaccharide on the reproductive functions of rabbit does. *Animal Reproduction Science*.

[25] Boiti C., Castellini C., Besenfelder U. (2005). Guidelines for the handling of rabbit bucks and semen. *World Rabbit Science*.

[26] Mourvaki E., Cardinali R., Dal Bosco A., Corazzi L., Castellini C. (2010). Effects of flaxseed dietary supplementation on sperm quality and on lipid composition of sperm subfractions and prostatic granules in rabbit. *Theriogenology*.

[27] Castellini C., Dal Bosco A., Ruggeri S., Collodel G. (2011). What is the best frame rate for evaluation of sperm motility in different species by computer-assisted sperm analysis?. *Fertility and Sterility*.

[28] Dal Bosco A., Mugnai C., Mourvaki E. (2009). Effect of genotype and rearing system on the native immunity and oxidative status of growing rabbits. *Italian Journal of Animal Science*.

[29] Signorini C., De Felice C., Durand T. (2018). Relevance of 4-F(4t)-neuroprostane and 10-F(4t)-neuroprostane to neurological diseases. *Free Radical Biology & Medicine*.

[30] Signorini C., Comporti M., Giorgi G. (2003). Ion trap tandem mass spectrometric determination of F2-isoprostanes. *Journal of Mass Spectrometry*.

[31] Folch J., Lees M., Sloane-Stanley G. H. (1957). A simple method for the isolation and purification of total lipids from animal tissues. *Journal of Biological Chemistry*.

[32] Christie W. W. (1982). A simple procedure for rapid transmethylation of glycerolipids and cholesteryl esters. *Journal of Lipid Research*.

[33] Mattioli S., Dal Bosco A., Maranesi M., Petrucci L., Rebollar P. G., Castellini C. (2019). Dietary fish oil and flaxseed for rabbit does: fatty acids distribution and Δ6-desaturase enzyme expression of different tissues. *Animal*.

[34] Castellini C., Dal Bosco A., Mattioli S. (2016). Activity, expression, and substrate preference of the ∆(6)-desaturase in slow- or fast-growing rabbit genotypes. *Journal of Agricultural and Food Chemistry*.

[35] Collodel G., Moretti E., Longini M., Pascarelli N. A., Signorini C. (2018). Increased F_2_-Isoprostane Levels in Semen and Immunolocalization of the 8-Iso Prostaglandin F_2 *α*_ in Spermatozoa from Infertile Patients with Varicocele. *Oxidative Medicine and Cellular Longevity*.

[36] STATA (2015). *Statistical Software, Version 14*.

[37] Perumal P., Chang S., Kobu K., Vupru K., Bag S. (2019). Flaxseed oil modulates semen production and its quality profiles, freezability, testicular biometrics and endocrinological profiles in mithun. *Theriogenology*.

[38] Castellano C. A., Audet I., Laforest J. P., Matte J. J., Suh M. (2011). Fish oil diets alter the phospholipid balance, fatty acid composition, and steroid hormone concentrations in testes of adult pigs. *Theriogenology*.

[39] Iizuka-Hishikawa Y., Hishikawa D., Sasaki J. (2017). Lysophosphatidic acid acyltransferase 3 tunes the membrane status of germ cells by incorporating docosahexaenoic acid during spermatogenesis. *Journal of Biological Chemistry*.

[40] Marzouki Z. M., Coniglio J. G. (1982). Effect of essential fatty acid deficiency on lipids of rat Sertoli and germinal cells. *Biology of Reproduction*.

[41] Hu X., Ge X., Liang W. (2018). Effects of saturated palmitic acid and omega-3 polyunsaturated fatty acids on Sertoli cell apoptosis. *Systems Biology in Reproductive Medicine*.

[42] Shultz T. D., Bonorden W. R., Seaman W. R. (1991). Effect of short-term flaxseed consumption on lignan and sex hormone metabolism in men. *Nutrition Research*.

[43] Qi X., Shang M., Chen C. (2019). Dietary supplementation with linseed oil improves semen quality, reproductive hormone, gene and protein expression related to testosterone synthesis in aging layer breeder roosters. *Theriogenology*.

[44] Li W., Tang D., Li F. (2017). Supplementation with dietary linseed oil during peri-puberty stimulates steroidogenesis and testis development in rams. *Theriogenology*.

[45] Gazouli M., Yao Z. X., Boujrad N., Corton C. J., Culty M., Papadopoulos V. (2002). Effect of peroxisome proliferators on Leydig cell peripheral-type benzodiazepine receptor gene expression, hormone-stimulated cholesterol transport, and steroidogenesis: role of the peroxisome proliferator-activator receptor *α*. *Journal of Endocrinology*.

[46] Conquer J. A., Martin J. B., Tummon I., Watson L., Tekpetey F. (2000). Effect of DHA supplementation on DHA status and sperm motility in asthenozoospermic males. *Lipids*.

[47] Safarinejad M. R. (2011). Effect of omega-3 polyunsaturated fatty acid supplementation on semen profile and enzymatic anti-oxidant capacity of seminal plasma in infertile men with idiopathic oligoasthenoteratospermia: a double-blind, placebo-controlled, randomised study. *Andrologia*.

[48] Butts I. A., Baeza R., Støttrup J. G. (2015). Impact of dietary fatty acids on muscle composition, liver lipids, milt composition and sperm performance in European eel. *Comparative Biochemistry and Physiology Part A: Molecular & Integrative Physiology*.

[49] Argov-Argaman N., Mahgrefthe K., Zeron Y., Roth Z. (2013). Season-induced variation in lipid composition is associated with semen quality in Holstein bulls. *Reproduction*.

[50] Ramos Angrimani D. S., Nichi M., Losano J. D. A. (2017). Fatty acid content in epididymal fluid and spermatozoa during sperm maturation in dogs. *Journal of Animal Science and Biotechnology*.

[51] Milne G. L., Yin H., Hardy K. D., Davies S. S., Roberts L. J. (2011). Isoprostane generation and function. *Chemical Reviews*.

[52] Castellini C., Dal Bosco A., Bernardini M. (1999). Effect of dietary vitamin E supplementation on the characteristics of refrigerated and frozen rabbit meat. *Italian Journal of Food Science*.

[53] Castellini C., Dal Bosco A., Bernardini M. (2001). Improvement of lipid stability of rabbit meat by vitamin E and C administration. *Journal of the Science of Food and Agriculture*.

[54] Halliwell B., Gutteridge J. M. (2015). *Free Radicals in Biology and Medicine*.

[55] Dasilva G., Medina I. (2019). Lipidomic methodologies for biomarkers of chronic inflammation in nutritional research: *ω*-3 and *ω*-6 lipid mediators. *Free Radical Biology and Medicine*.

[56] Montine T. J., Quinn J. F., Milatovic D. (2002). Peripheral F2-isoprostanes and F4-neuroprostanes are not increased in Alzheimer’s disease. *Annals of Neurology*.

[57] Reich E. E., Markesbery W. R., Roberts L. J., Swift L. L., Morrow J. D., Montine T. J. (2001). Brain Regional Quantification of F-Ring and D-/E-Ring Isoprostanes and Neuroprostanes in Alzheimer's Disease. *American Journal of Pathology*.

[58] Comporti M., Signorini C., Arezzini B., Vecchio D., Monaco B., Gardi C. (2008). F 2-isoprostanes are not just markers of oxidative stress. *Free Radical Biology and Medicine*.

[59] Zaja-Milatovic S., Gupta R. C., Aschner M., Milatovic D. (2009). Protection of DFP-induced oxidative damage and neurodegeneration by antioxidants and NMDA receptor antagonist. *Toxicology and Applied Pharmacology*.

[60] Roberts L. J., Milne G. L. (2009). Isoprostanes. *Journal of Lipid Research*.

[61] Gao L., Yin H., Milne G. L., Porter N. A., Morrow J. D. (2006). Formation of F-ring isoprostane-like compounds (F3-isoprostanes) in vivo from eicosapentaenoic acid. *Journal of Biological Chemistry*.

[62] Syta-Krzyżanowska A., Jarocka-Karpowicz I., Kochanowicz J. (2018). F2-isoprostanes and F4-neuroprostanes as markers of intracranial aneurysm development. *Advances in Clinical and Experimental Medicine*.

[63] Serhan C. N., Chiang N. (2008). Endogenous pro-resolving and anti-inflammatory lipid mediators: a new pharmacologic genus. *British Journal of Pharmacology*.

